# CAREGIVER AND VETERAN PERCEPTIONS OF THE REFINING ELDER CARE IN AMERICA PROGRAM (RECAP)

**DOI:** 10.1093/geroni/igad104.0164

**Published:** 2023-12-21

**Authors:** Leah Haverhals, Kate Magid, Mary Nunnery, Chelsea Manheim, Jessica Young, Cari Levy

**Affiliations:** Department of Veterans Affairs Rocky Mountain Regional VA Medical Center, Denver, Colorado, United States; Veterans Health Administration, Aurora, Colorado, United States; Rocky Mountain Regional VAMC, Aurora, Colorado, United States; Rocky Mountain Regional VAMC, Aurora, Colorado, United States; VA Puget Sound Health Care System, Seattle, Washington, United States; University of Colorado, Denver, Colorado, United States

## Abstract

In 2021, the Veterans Health Administration (VHA) piloted the Redefining Elder Care in America Program (RECAP) at two Department of Veterans Affairs (VA) health care centers. RECAP identifies Veterans at risk for nursing home placement, and coordinators conduct a phone assessment with Veterans or their caregivers to link Veterans to VA services. To identify benefits and challenges of RECAP from Veterans’ and caregivers’ perspectives, we conducted phone interviews (N=32) from October 2021- May 2022. We analyzed data using a thematic approach, finding that participants appreciated the proactive nature of RECAP, felt supported by RECAP coordinators, and felt the VA cared about them. Caregivers cited RECAP’s benefits, such as learning about VA services and having the RECAP coordinator as a point of contact to help them navigate the VA. Caregivers of Veterans who received services reported improvements in caregiver mental health, decreases in caregiver burden, and improvements in Veterans’ abilities to remain at home. A few caregivers were uncertain if they could trust the RECAP phone assessment, thinking it was a scam, and two reported challenges contacting the coordinator after the assessment. Several caregivers expressed uncertainty about the timeline for service delivery and next steps to obtain services following the assessment. Caregivers described challenges related to receiving services including Veterans not being eligible for the chosen service and receiving fewer hours of home health care than desired. Overall, participants were satisfied with RECAP. Challenges identified should be addressed to best meet the needs of Veterans and Caregivers.

